# The Scr and Csc pathways for sucrose utilization co-exist in *E. coli*, but only the Scr pathway is widespread in other *Enterobacteriaceae*

**DOI:** 10.3389/fmicb.2024.1409295

**Published:** 2024-07-03

**Authors:** Craig Stephens, Mireille Martinez, Virginia Leonardi, Jasmine Jaing, Anna Miller

**Affiliations:** Department of Biology, Santa Clara University, Santa Clara, CA, United States

**Keywords:** *Escherichia coli*, sucrose, evolutionary genomics, metabolism, Scr, Csc

## Abstract

Most *Escherichia coli* isolates from humans do not utilize D-sucrose as a substrate for fermentation or growth. Previous work has shown that the Csc pathway allows some *E. coli* to utilize sucrose for slow growth, and this pathway has been engineered in *E. coli* W strains to enhance use of sucrose as a feedstock for industrial applications. An alternative sucrose utilization pathway, Scr, was first identified in *Klebsiella pneumoniae* and has been reported in some *E. coli* and *Salmonella enterica* isolates. We show here that the Scr pathway is native to an important subset of *E. coli* phylogroup B2 lineages that lack the Csc pathway but grow rapidly on sucrose. Laboratory *E. coli* strains derived from MG1655 (phylogroup A, ST10) are unable to utilize sucrose and lack the *scr* and *csc* genes, but a recombinant plasmid-borne *scr* locus enables rapid growth on and fermentation of sucrose. Genome analyses of *Enterobacteriaceae* indicate that the *scr* locus is widespread in other *Enterobacteriaceae*; including *Enterobacter* and *Klebsiella* species, and some *Citrobacter* and *Proteus* species. In contrast, the Csc pathway is limited mostly to *E. coli*, some *Shigella* species (in which *csc* loci are rendered non-functional by various mutations), and *Citrobacter freundii*. The more efficient Scr pathway likely has greater potential than the Csc pathway for bioindustrial applications of *E. coli* and other *Enterobacteriaceae* using sucrose as a feedstock.

## Introduction

D-Sucrose, a disaccharide of glucose and fructose, is common in the biosphere due to its production in many plant tissues. It is a major product of human agriculture and can be advantageous for microbially-mediated production of alcohols and other industrially-relevant chemicals (Peters et al., 2010). However, the ability to utilize sucrose as a carbon and energy source is not universal among microbes, and is highly variable among Gram-negative bacteria of the family *Enterobacteriaceae* (Le Bouguénec and Schouler, 2011). For example, isolates of *Salmonella* and *Shigella* rarely utilize sucrose, but isolates of *Enterobacter* and *Klebsiella* usually do. In our experience, roughly one third of clinical and commensal *E. coli* isolates ferment sucrose in the API20E rapid identification system (Holmes et al., 1978). We describe here the genomic basis for variability in sucrose utilization in *E. coli*, *Shigella*, and other *Enterobacteriaceae*.

**Figure 1:**
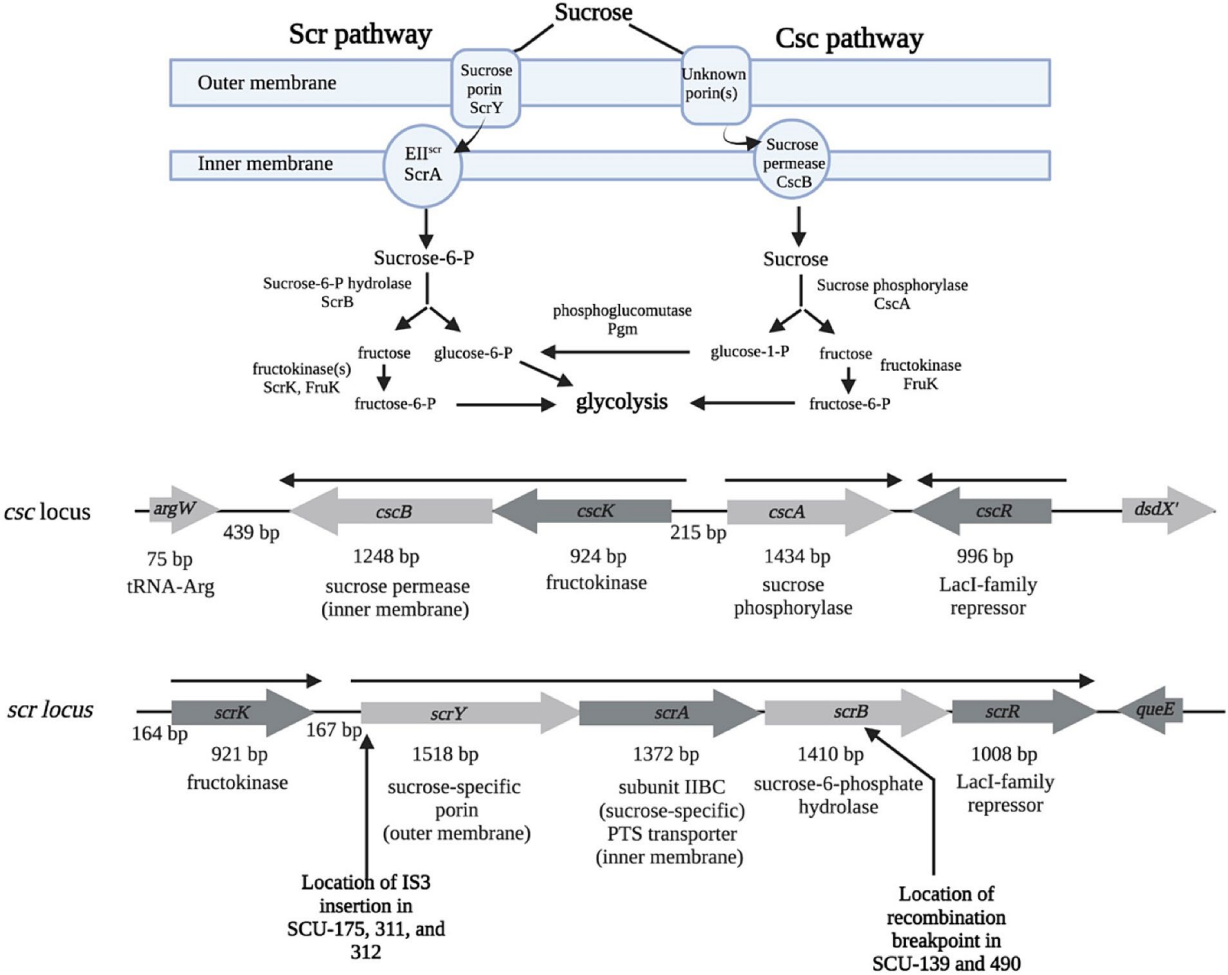
Function and genetic organization of the Scr and Csc pathways for sucrose utilization. Inferred structure and function of the Scr and Csc pathways (reviewed in Reid and Abratt, 2005). Organization of the *E. coli* genetic loci encoding the Scr and Csc pathways, and the regulators controlling them (*cscR* and *scrR*, respectively). The transcriptional organizations of the loci are shown in solid arrows above the genes. Approximate locations of IS3 insertions (*scrY*) and recombination event (*scrB*) discussed in Results are indicated below the *scr* locus. Figure created using BioRender.

Variability in sucrose utilization within *Enterobacteriaceae* was initially suggested to be due to carriage of key genes on plasmids, with Wohlhieter et al. (1975) observing that a rare sucrose-utilizing *Salmonella* isolate could conjugally transfer the phenotype to *E. coli*, and Palchaudhuri et al. (1977) reporting a sucrose-utilizing *E. coli* isolate in which the Suc^+^ trait was apparently carried on a non-conjugal plasmid. An *S. typhimurium* plasmid, pUR400, conveying the Suc^+^ phenotype was subsequently characterized (Schmid et al., 1982; García, 1985). Analysis of the plasmid-based *Salmonella* system (Schmid et al., 1988), along with the homologous chromosomally-encoded *Klebsiella pneumoniae* system (Sprenger and Lengeler, 1988; Titgemeyer et al., 1996) brought the details of the *scr* pathway into focus (Figure 1). Sucrose is brought into the periplasm via a sucrose-specific outer membrane porin, ScrY (Hardesty et al., 1991; Schmid et al., 1991; Schülein et al., 1991). From the periplasm, sucrose crosses the cytoplasmic membrane via a PEP-dependent transport (PTS) system, with ScrA comprising the sucrose-specific EII^scr^ membrane channel component (Lengeler et al., 1982; Schmid et al., 1982). In the cytoplasm, sucrose-6-phosphate is cleaved by sucrose-6-phosphate hydrolase (ScrB) into fructose and glucose-6-phosphate (Schmid et al., 1982). G-6-P is fed into glycolysis, and fructose is phosphorylated to fructose-6-phosphate by fructokinase (ScrK) (Aulkemeyer et al., 1991).

As the *scr* genes were being explored, Alaeddinoglu and Charles (1979) found that sucrose utilization can be a chromosomally-encoded trait in some *E. coli*, where it was mutually exclusive with D-serine utilization. Bockmann et al. (1992) found that *E. coli* EC3132 could process sucrose through a pathway they designated Csc (“*c*hromosomally-coded *s*u*c*rose genes”) (Figure 1; Bockmann et al., 1992). There is no dedicated porin component in the Csc system, so non-specific transport of sucrose into the periplasm via one or more constitutive porin(s) is assumed. Cytoplasmic membrane transport is via the CscB sucrose permease, a non-PTS sucrose-H^+^ symporter homologous to LacY (Bockmann et al., 1992). In the cytoplasm, sucrose is cleaved by sucrose phosphorylase (CscA) into glucose-1-phosphate and fructose. The *csc* locus also includes a LacI-type repressor (CscR) that controls expression of the *csc* genes in response to sucrose; the genes also respond to catabolite repression via cAMP and CAP (Bockmann et al., 1992). As Jahreis et al. (2002) noted, growth of EC3132 on sucrose as the sole carbon and energy source is slow, leading to efforts to increase growth rates by mutation and selection in both EC3132 and *E. coli* W (Jahreis et al., 2002; Bruschi et al., 2012; Sabri et al., 2013). Enhanced growth rates on sucrose have been achieved via mutations derepressing *csc* expression by targeting the interaction of CscR with its operator target(s), or by mutationally increasing transport flux through CscB (Jahreis et al., 2002; Sabri et al., 2013).

We explore here the genomic basis for sucrose utilization, or lack thereof, in diverse *E. coli* lineages, in *Shigella* species, and in related *Enterobacteriacea*, finding that the Scr and Csc pathways show starkly contrasting evolutionary histories. These insights not only shed light on the evolutionary history of sucrose utilization in this group of bacteria, but in practical terms may encourage more effective engineering of *E. coli* for bioindustrial applications with sucrose-rich organic materials as feedstock.

## Materials and methods

### Bacterial strains and media

Commensal *E. coli* isolates used in this work were isolated from healthy college students, as described in Stephens et al. (2020). Bacteria were routinely cultured on LB broth or agar. Utilization of sugars as sole carbon and energy sources was tested by growth on M9 minimal salts medium, with the designated sugar at a starting concentration of 10 mM. Plates or liquid media containing D-glucose are referred to as “M9G,” while those containing D-sucrose are referred to as “M9S” herein. Cultures were incubated at 37°C (with shaking for liquid cultures). Fermentation was tested by growth in phenol red broth (Hardy Diagnostics) with the designated sugar present at a starting concentration of 0.5%. Cultures were incubated at 37°C with no shaking.

### Bioinformatic analysis

The genome sequences of commensal *E. coli* used herein have been described previously (Stephens et al., 2020). Isolates were assigned to MLST groups using the web-based MLST 2.0 algorithm (Larsen et al., 2012). BLAST/MegaBLAST searches (Altschul et al., 1990) of these genomes, as well as basic molecular biological processes such as PCR primer design, were done locally using the Geneious Prime desktop bioinformatics package (Dotmatics). Searches of the RefSeq database (Pruitt et al., 2007) were done with NCBI BLAST (Johnson et al., 2008). *scr* genes from the closest known related species were used as queries, to compensate for evolutionary drift between gene sequences from more distant relatives. To identify *E. coli* genes uniquely associated with sucrose utilization, a subset of commensal *E. coli* genomes of known sucrose phenotypes were submitted to the Genomes Online Database (GOLD; Mukherjee et al., 2023). The Phylogenetic Profiler tool within the Integrated Microbial Genomes and Microbiomes platform (IMG/G; Joint Genome Institute, Walnut Creek, USA) (Chen et al., 2023) was then used for analysis.

### Cloning and expression of *scr* locus

Genomic DNA was extracted using the NucleoSpin microbial DNA purification kit (Macherey-Nagel). For amplification of the *scr* genes by polymerase chain reaction (PCR), primer pairs for the target gene regions were designed using Geneious bioinformatics software (Biomatters LTD), and synthesized by Integrated DNA Technologies (Alameda, CA, USA). The entire 6.6 kb *scr* locus was amplified using primers *scrK*43F (TCC CGG CAT ATT CAC GTT TCC AC) and *scrR*6689R (CCG TTT TAC AGG GGC GAT GCA). The *scrY* gene alone was amplified using primers *scrY*1058F (ACC GCC TTA CCC CGA CAA CA) and *scrA*2818R (TTC AGT AAA AGC CTC ACA TCC GT). Genomic DNA from *E. coli* strain SCU-147 (Stephens et al., 2020) was used as a template for amplification of the *scr* genes for cloning. Products of appropriate sizes were verified by agarose gel electrophoresis, then prepared for cloning using the Gel and PCR Clean-up kit (Macherey-Nagel). The PCR Cloning Kit (New England Biolabs) was used to clone amplicons into plasmid pMiniT2.0, and ligated DNA was electroporated into *E. coli* NEB 10-beta C3019H, with selection on LB agar containing 100 μg/mL ampicillin. Four colonies were selected from each ligation and transformation and used for colony PCR. The same primers were used to assess putative recombinant plasmids. Colonies that produced strong product bands of the target size were grown overnight in liquid LB with 100 μl/mL ampicillin, and plasmid DNA was prepared using a commercial kit (Zymo). Plasmid DNA was digested with restriction enzyme EcoRI to verify expected insert size, and candidate clones were further verified by Sanger sequencing (Sequetech, Mountain View, CA, USA) to confirm the expected insert. Plasmid DNA was subsequently used to transform natural commensal *E. coli* isolates from Stephens et al. (2020), prepared by calcium treatment (Chang et al., 2017). To remove *scrR* from the *scr* locus, pMM007 was digested with BamHI, resulting in a 7.7 kb fragment product (pMM008). A partial internal 500 bp fragment of *scrY* was removed by digestion with EcoRV. The larger linearized plasmid was re-ligated with T4 DNA ligase using quick T4 DNA ligase (New England Biolabs), then transformed into NEB 10-beta C3019H.

## Results

### Utilization of D-sucrose by commensal *Escherichia coli* isolates

**Figure 2:**
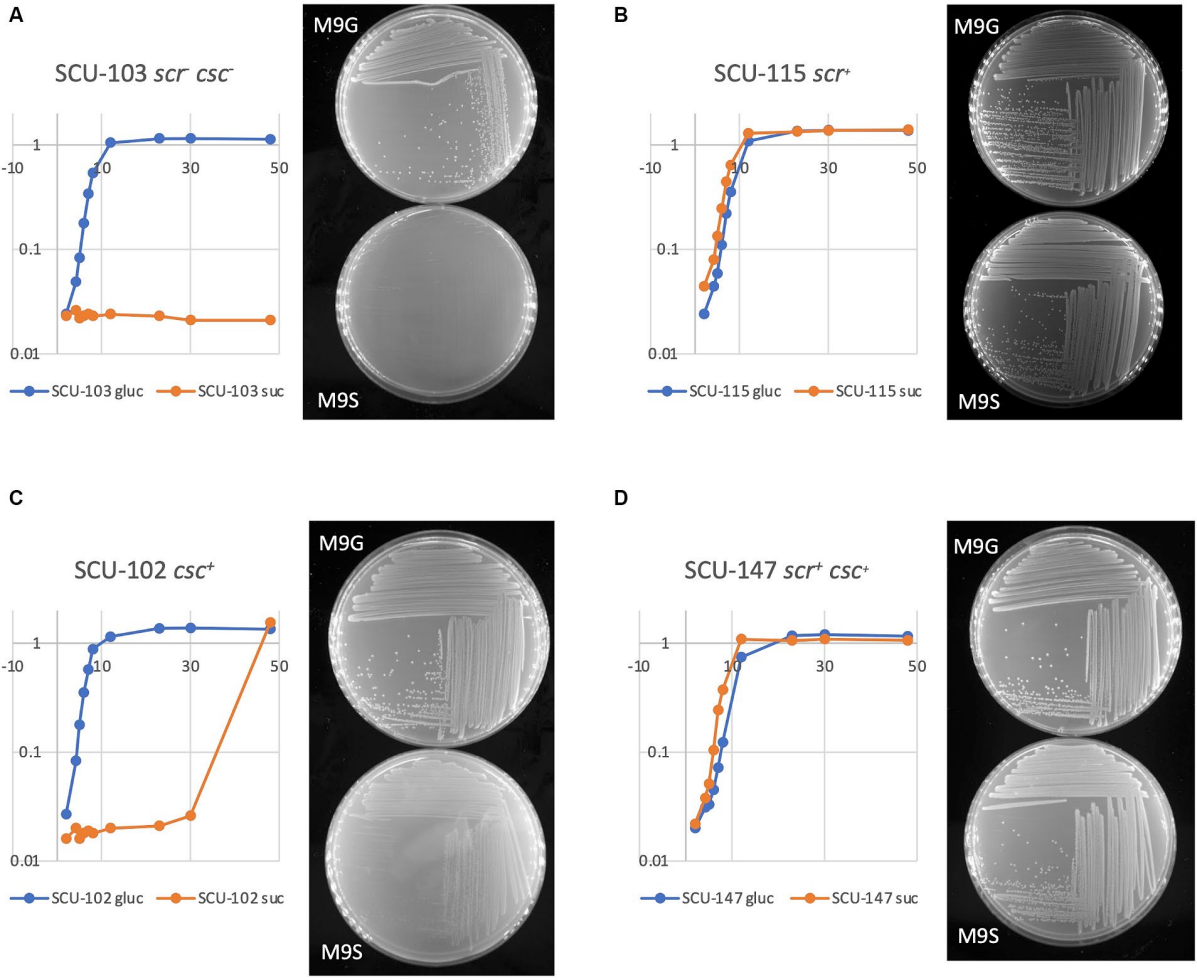
Assays for sucrose utilization in liquid and agar media. Culture growth in M9 minimal salts broth with either glucose or sucrose as sole carbon/energy source was monitored in a spectrophotometer at 600 nm, whom on the left of each panel. M9 agar plates with either glucose (M9G) or sucrose (M9S) were photographed 24 h after streaking.

To explore the genetic and physiological basis for sucrose utilization in *E. coli*, we employed a collection of diverse commensal *E. coli* strains with sequenced genomes (Stephens et al., 2020). The collection includes more than 100 representatives of all major *E. coli* phylogroups (A, B1, B2, C, D, E, and F) (Denamur et al., 2021). Table 1 shows results for a limited subset of 32 isolates, each representing a different MLST group, and each with fully assembled, closed genomes available in GenBank. Broth and agar plate-based assays were used to examine growth using sucrose as sole carbon and energy source. Representative growth curves and images of M9G and M9S plates are shown in Figure 2. The “Suc^+^” phenotype (Figures 2B,D) was defined here as being able to utilize D-sucrose as sole carbon and energy source in M9 minimal salts medium at a growth rate similar to glucose; under the conditions applied (small test tube cultures grown in a shaking incubator at 37°C), this corresponded to a growth rate *μ* of 0.8–1.1 generations/h. Roughly 20% of commensal *E. coli* strains in our collection were Suc^+^ (6/32 shown in Table 1). A slightly higher fraction (9/32, Table 1) were able to more slowly utilize D-sucrose for growth (Figure 2C and Table 1; isolates with *μ* = 0.4–0.7 gen h^−1^ were designated “slow,” isolates with *μ* = 0.1–0.3 were designated “very slow” growth). The growth rate of colonies on M9S for these isolates was noticeably slower (Figure 2C). The remaining isolates (17/32 in Table 1) were unable to use D-sucrose as the sole carbon and energy source for significant growth (Suc^−^, Figure 2A).

**Table 1:** Commensal *E. coli* sucrose utilization phenotypes and genotypes.

Phylo-group	MLST	Representative isolate (GenBank accession #)	Growth on glucose^1^	Growth on sucrose^1^	*scr locus*	*csc locus*	Comments
B2	491	SCU-111 CP051727	+	−	−	−	
	95	SCU-108 CP051735	−	−	−	−	Basis for lack of growth on glucose unknown
	114	SCU-125 CP051700	+	−	−	−	
	73	SCU-112 CP051725	Slow	−	−	−	Basis for slow growth on glucose unknown
	1,262	SCU-101 CP051849	Slow	−	−	−	Basis for slow growth on glucose unknown
	1,193	SCU-147 CP054325	+	+	+	+	
	550	SCU-176 CP054345	Very slow	Very slow	+	+	Basis for very slow growth on both substrates unknown
	14	SCU-387 CP051688	+	+	+	+	
	91	SCU-121 CP054328	+	+	+	−	
	1,155	SCU-115 CP054368	+	+	+	−	
	357	SCU-124 CP051706	+	+	+	−	
	131	SCU-182 CP054372	+	+	+	−	
	2,279	SCU-479 CP054317	+	Slow	+	−	Basis for slow growth on sucrose unknown
	657	SCU-171 CP054363	Slow	−	−	+	Basis for slow growth on glucose, lack of growth on sucrose unknown
D	69	SCU-313 CP051694	+	Slow	−	+	
	394	SCU-105 CP051738	+	Very slow	−	+	
	38	SCU-397 CP054828.1	+	Very slow	−	+	
	963	SCU-109 CP051733	+	Very slow	−	+	
	973	SCU-102 CP051753	+	Very slow	−	+	
F	62	SCU-175 CP054379.1	Slow	−^2^	+	−	Basis for slow growth on glucose unknown; Is3 insertion in *scrY* likely causes lack of growth on sucrose
	379	SCU-172 CP054353	+	−	−	−	
	967	SCU-301 CP051751	+	−	−	−	
	648	SCU-120 CP054335	+	−	−	−	
A	8,125	SCU-104 CP053284	+	−	−	−	
	10	SCU-103 CP054457	+	−	−	−	
B1	54	SCU-152 CP051698	+	−	−	+	Basis for lack of growth on sucrose unknown
	5,974	SCU-113 CP051765	+	−	−	−	
	48	SCU-308 CP053281	Slow	Very slow	−	+	Basis for slow growth on glucose unknown
	10,955	SCU-483 CP054314	Slow	−	−	+	Basis for slow growth on glucose, lack of growth on sucrose unknown
	164	SCU-478 CP054564.1	+	Slow	−	+	
	3,695	SCU-106 CP05B1/ST54)4	+	−	−	+	10 bp insertion in *cscB* causes frameshift, likely responsible for lack of growth on sucrose
E	57	SCU-316 CP054371	+	−	−	+	Basis for lack of growth on sucrose unknown

^1^Classification based on growth on M9 minimal medium at 37°C in 13 mm test tubes with shaking, measured by OD_600 nm_, with growth rate (*μ*) calculated during log phase. “+”, *μ* = 0.8–1.1 gen/h; “slow”, *μ* = 0.4–0.7 gen/h; “very slow”, 0.1–0.3; “−”, *μ* < 0.1 gen/h.

^2^Both plate and liquid growth patterns showed no detectable growth on M9 sucrose media for 12 h or more, followed by the subsequent appearance of genetically-stable suc^+^ cells/colonies that upon isolation were able to grow at “normal” rates on sucrose.

### Genetic basis of sucrose utilization

To identify genes associated with the Suc^+^ phenotype, fully assembled and annotated genomes of four Suc^+^ isolates (SCU-115, phylogroup B2, MLST type ST1155; SCU-147, B2/ST1193; SCU-182, B2/ST131; SCU-387, B2/ST14) were compared to the genomes of eight phylogenetically diverse Suc^−^ isolates (SCU-103, A/ST10; SCU-152, B1/ST54; SCU-108 B2/ST95; SCU-112 B2/ST73; SCU-321, D/ST69; SCU-106, E/ST3695; SCU-120 F/ST648, and SCU-301 F/ST967) using the Phylogenetic Profiler tool of the IMG/G platform. Just five genes (*scrA*, *scrB*, *scrK*, *scrR*, and *scrY*) were present in the genomes of all four Suc^+^ isolates (minimum 80% identity) and absent in all of the genomes of the eight Suc^−^ isolates. The *scr* genes are clustered in two adjacent transcriptional units, *scrK* and *scrYABR* (Cowan et al., 1991). The 6.7 kb *scr* locus was highly conserved, with ≥99% identity among the four Suc^+^ isolates.

### A recombinant plasmid-borne *scr* locus enables growth on sucrose

**Figure 3:**
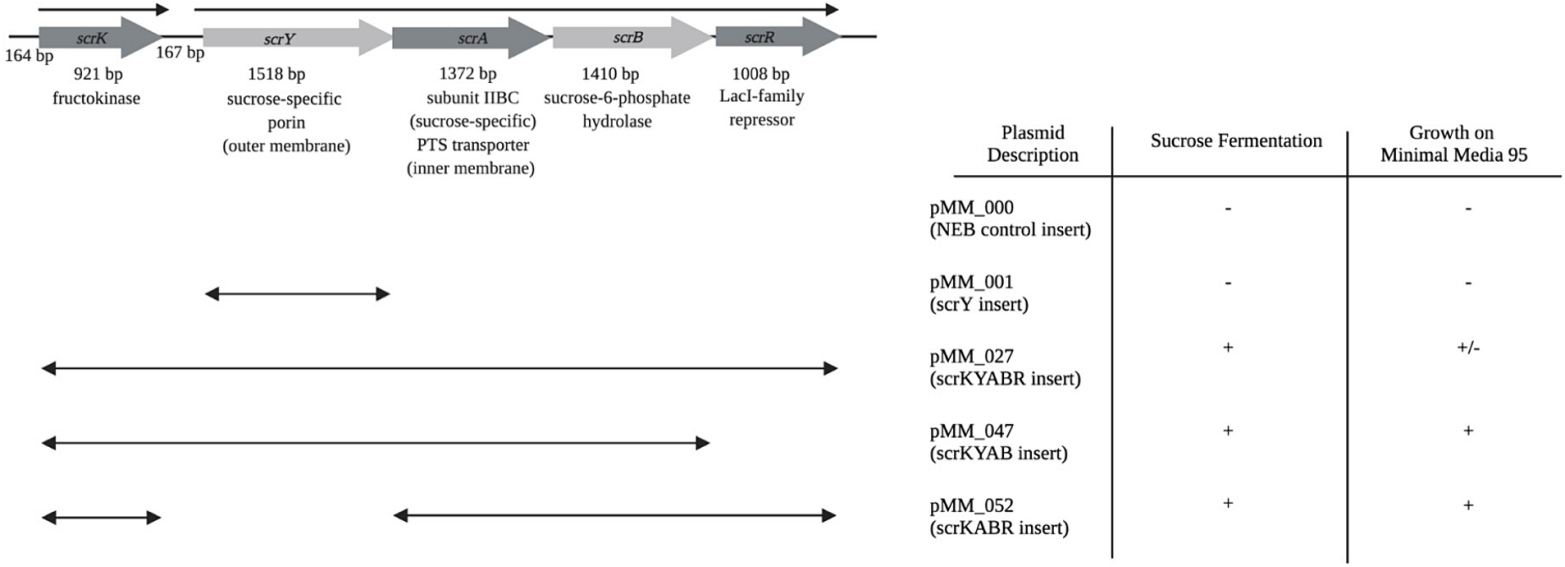
Utilization of sucrose conferred by recombinant plasmids with *scr* genes. Life side of the figure diagrams inserts in recombinant plasmids (named on the right side) containing the entire *scr* operon, or fragments thereof. Plasmids were introduced into *E. coli* SCU-113 (B1, ST5974, *scr*^−^ *csc*^−^). In the table, “+” indicates that fast fermentation/growth was observed, “+/−” indicates that slower fermentation and growth was observed, and “−” indicates that no fermentation/growth was observed.

To test the sufficiency of the *scr* locus for sucrose utilization, the region was amplified by PCR and cloned into plasmid vector pMiniT2.0 (Figure 3). The recombinant *scr^+^* plasmid construct pMM_027 was introduced into the *E. coli* NEB10-beta strain, a derivative of the standard DH10B cloning strain, which is in turn derived from *E. coli* MG1655 (Durfee et al., 2008), a phylogroup A/ST10 strain. Phylogroup A *E. coli* in our collection were universally unable to use sucrose, and lack the *scr* or *csc* loci. Neither the parental NEB10-beta strain or the recombinant NEB10-beta/pMM_027 strain were able to grown on M9 minimal medium with glucose or sucrose as sole carbon and energy source, presumably due to auxotrophies resulting from the complex genotype of NEB10b. They were, however, able to ferment glucose in phenol-red broth. NEB10-beta/pMM_027 gained the ability to rapidly ferment sucrose in phenol red broth. When pMM_027 was moved into SCU-113 (B1, ST5974, *scr*^−^ *csc*^−^), a prototrophic *E. coli* commensal isolate, the resulting strain was able to both ferment sucrose and utilize sucrose as sole carbon and energy source on M9 medium, albeit more slowly than a native Suc^+^ B2 strain. These results indicate that the *scr* locus is sufficient to convey the Suc^+^ phenotype.

Removal of the putative *scrR* transcriptional regulator from the recombinant construct (pMM_047) accelerated growth and fermentation of sucrose, as did deletion of the porin-encoding *scrY* gene (pMM_052). The *scrY* deletion removed the *scrYABR* operon promoter, but we speculate that it is possible that read-through transcription from *scrK* in the deletion construct provided sufficient expression of *scrA* and *scrB* to allow sucrose fermentation and growth.

### Phylogenetic distribution of *scr* locus

The sequenced commensal *E. coli* genomes in our collection were queried via BLAST for the *scr* locus (Table 1). The Suc^+^ phenotype and *scr* are largely associated with a branch of the B2 clade that includes the ST1193 and ST131 MLST groups, known for their extraintestinal virulence (Pitout et al., 2022). Overall, *scr* was found in nearly half of B2 isolates.

**Figure 4:**
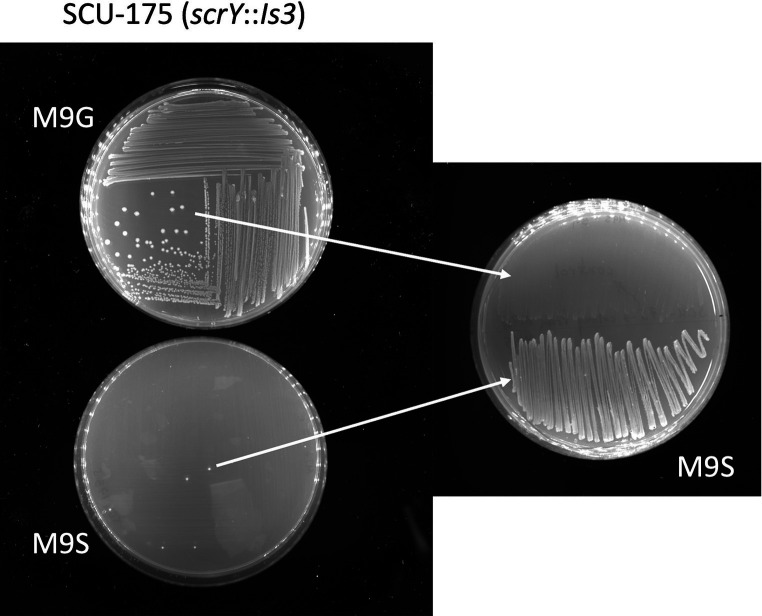
Rare spontaneous conversion of Suc^−^ SCU-175 (*scrY::Is3*) to Suc^+^ phenotype. The left panel shows that SCU-175 showed no growth on M9S plates after 48 h, with the exception of rare spontaneous colonies. When those colonies were restreaked on M9S plates (right panel, bottom half of plate) they were Suc^+^, in contrast to the parental strain streaked on the same plate as a control (right panel, top half).

The presence of the *scr* locus correlated well with the Suc^+^ phenotype, as the majority of *scr*-containing isolates were Suc^+^ (6/9 in Table 1, and 20/24 in our larger sequenced *E. coli* collection). Three unusual *scr*-containing isolates (SCU-175, 311, 312) from outside the B2 clade showed a Suc^−^ phenotype when incubated on M9S plates or liquid media for up to 1 day. However, isolated colonies did appear over time on these plates (SCU-175 is shown as an example in Figure 4). Genome sequence analysis revealed identical 1.9 kb *Is3* insertions located 12 bp into the *scrY* gene (Figure 1). This insertion is expected to knock out transport by ScrY, the sucrose-specific porin of the Scr system (Hardesty et al., 1991; Schmid et al., 1991). The pMM_052 recombinant construct discussed above showed that *scrY* is not strictly necessary for the Suc^+^ phenotype, but the *Is3* insertion in *scrY* may also have negative polar effects on expression of the downstream *scrA*, *scrB*, and *scrR* genes. GenBank searches identified four other examples of this precise configuration in *E. coli* F/ST62 isolates from around the world, but the *Is3* insertion is not universal in F/ST62 isolates. In fact, the closely-related SCU-114 isolate in our collection has an intact *scr* locus and is Suc^+^ (data not shown). As shown in Figure 4, the isolated colonies selected from prolonged incubation of *scrY::Is3* strains on M9 sucrose media stably gain the ability to utilize sucrose, as would be expected if the Is3 element has been excised.

Some *scr*^+^ isolates examined were not fully Suc^+^; for example, SCU-479 grew slowly, and SCU-176 grew very slowly on M9 sucrose. The basis for these phenotypes is not yet known, although we also noted that SCU-176 grew very slowly on glucose as well, so the growth phenotype may not be attributable specifically to a sugar utilization pathway.

Among 79 isolate genomes in our collection initially determined to lack *scr* (using the SCU-147 *scr* locus as a BLAST query), 54 isolates were Suc^−^, 26 were Suc^slow^, and two were Suc^+^: SCU-391 (A, ST216) and SCU-490 (B1, ST6496). Analysis of the SCU-391 and SCU-490 genomes did not reveal hits above 80% identity to the SCU-147 *scr* locus. However, further inspection showed that these genomes contained a more distantly related *scr* locus (~67% identity with SCU-147 *scr* over 6.7 kb). The SCU-391 *scr* locus closely matches SCU-490 for the *scrKYA* genes, but they diverge at codon 229 of *scrB*, continuing into *scrR* (Figure 1). The *scrKYA* genes of SCU-391/490 were used as queries to search GenBank, identifying closely related loci (>95% identity, >95% query coverage) in more than 200 *E. coli* RefSeq genomes (0.8% of the total). Strikingly similar (>99.7% identity, 100% query coverage) *scr* loci were found in 230/494 (47%) *C. freundii* RefSeq genomes, and in the genomes of several other non-*freundii Citrobacter*, *Enterobacter, and Klebsiella* isolates in GenBank. The divergent SCU-391 *scrBR* region was found in genomes of distinct *C. freundii* isolates. It therefore seems likely that *C. freundii* or a closely related species was the ancestral source of the *scr* loci in SCU-391 and 490, via independent transfers to A and B1 *E. coli*. Phenotypic analysis indicated that these *scr* loci are fully functional in *E. coli*; indeed, growth curve analysis of SCU-391 found that it grew more rapidly on sucrose than glucose (*μ*_suc_ = 1.15 gen h^−1^ vs. *μ*_gluc_ = 0.85 gen h^−1^) and that it’s growth rate on sucrose is higher than B2 isolates with the native scr locus (*μ*_suc_ = 0.8–1.1 gen h^−1^).

In *E. coli* SCU-147 and nearly all of the other *scr*^+^ *E. coli* B2 and F isolates in our collection with fully closed genomes, the *scr* locus is positioned on the chromosome adjacent to the *queE* gene. As noted by Díez-Villaseñor et al. (2010), in *scr*^−^ *E. coli* strains this is the location of the CRISPR2.2 array of CRISPR2 repeats, also known as *iap* repeats (Treviño-Quintanilla et al., 2007). The *queE*-CRISPR2.2 region is roughly 25 kb from the region encoding the CRISPR-CAS-E system in *E. coli* with the CAS-E genes. The 6.7 kb *scr* locus replaces approximately 0.5 kb of non-homologous DNA adjacent to *queE*. No IS or transposable elements flanking *scr* were noted that might mechanistically account for the insertion. In SCU-391 the *C. freundii*-like *scr* locus was located nearly on the opposite side of the chromosome, adjacent to a prophage genome resembling *Shigella* SFII. It appears likely that the *C. freundii*-like *scr* locus has been horizontally transferred among Gram-negative enterobacterial species. Indeed, GenBank searches suggest that many of these *C. freundii*-like *scr* loci in *E. coli* genomes reside on plasmids (data not shown), the chromosomal location in SCU-391 notwithstanding.

For a more historic view of the *scr* locus in *E. coli*, genomes of strains in the ECOR collection (Ochman and Selander, 1984) were examined. This collection was assembled to reflect the genetic diversity of *E. coli* as understood in the mid- to late 20th century. The *scr* locus was present in 8/72 of the ECOR collection genomes (11%), lower than the 21% in our contemporary commensal collection, which is significantly richer in B2 isolates. The *scr* locus is found in 4/16 (25%) of the ECOR B2 strains and 4/6 (67%) of the F strains (all intact *scr* loci). No other versions of the *scr* genes, such as the *C. freundii*-like *scr*, were detected in the ECOR genomes.

*Shigella* species have emerged evolutionarily multiple times from within the broader *E. coli* lineage (The et al., 2016), but clinical diagnostic databases such as API20E indicate that *Shigella* isolates rarely if ever ferment sucrose. The presence of the *scr* locus in *Shigella* genomes was queried in the RefSeq genome database. The *scr* genes were very rare, being present in less than 1% of sequenced *S. sonnei* and *S. flexneri* genomes, and in no *S. dysenteriae* or *S. boydii* genomes.

Beyond *E. coli* and *Shigella*, sucrose utilization is variable within the family *Enterobacteriaceae*. Data on sucrose fermentation from the API 20E database was used as a surrogate phenotypic assay for comparison with genome-based surveys of the *scr* locus in the RefSeq database. Results are shown in Table 2 for the *scrA* locus. The presence of *scrA* was correlated with sucrose utilization by species. For example, less than 1% of *Salmonella* isolates ferment sucrose, and in the *Salmonella enterica* RefSeq database of over 10,000 genomes, only eight contained the entire *scr* locus (minimum 80% coverage, 80% nucleotide identity). A rare exception to the exclusive role of the Scr pathway in sucrose utilization was seen in *Citrobacter freundii*. Isolates of this species near universally ferment sucrose, but only half of the *C. freundii* genomes in RefSeq contain the *scr* genes; however, as described below, the alternative *csc* locus is ubiquitous in *C. freundii*. Other *Citrobacter* species rarely or never utilize sucrose, and the *scr* and *csc* genes are concomitantly scarce. Conversely, *Enterobacter* and *Klebsiella* species almost always ferment sucrose, and nearly all of those genomes in the RefSeq collection contain the *scr* locus. *Proteus vulgaris* isolates almost all ferment sucrose and contain the *scr* locus, but *P. mirabilis* isolates rarely do either. Moving beyond the *Enterobacteriaceae* to related families in the order *Enterobacterales*, the *scr* locus is near universal in *Erwinia amylovora*, *Serratia marcescens*, and *Yersinia enterocolitica*, but *Y. pestis* and *Y. pseudotuberculosis* rarely ferment sucrose or contain *scr* genes.

**Table 2:** Phylogenetic distribution of Scr and Csc pathways for sucrose metabolism.

Family	Genus	Representative species	Suc^+^ in API20E reference data (%)	RefSeq genomes with *scrA* (%)	RefSeq genomes with *cscA* (%)
*Enterobacteriaceae*	*Escherichia*	*E. fergusoni*	0	0	1
		*E. albertii*	ND^1^	12	0
	*Shigella*	*S. sonnei*	1	<1	>99
		*S. flexneri*	1	<1	70
		*S. dysenteriae*	ND	0	41
		*S. boydii*	ND	0	2
	*Citrobacter*	*C. freundii*	99	49	95
		*C. amalonaticus*	1	5	0
		*C. koseri*	ND	21	0
	*Salmonella*	*S. enterica*	0	<1	<1
	*Enterobacter*	*E. cloacae*	96	100	0
		*E. hormaechii*	ND	85	0
	*Klebsiella*	*K. oxytoca*	99	>99	0
		*K. pneumoniae*	99	>99	1
	*Cedecea*	*C. davisae*	100	71	0
	*Proteus*	*P. mirabilis*	1	<1	0
		*P. vulgaris*	89	>99	0
*Erwiniaceae*	*Erwinia*	*E. amylovora*	ND	>99	0
*Yersiniaceae*	*Serratia*	*S. marcescens*	99	94	0
		*S. rubidaea*	99	92	0
	*Yersinia*	*Y. enterocolitica*	99	>99	0
		*Y. pestis*	0	0	0
		*Y. pseudotubercul.*	0	0	0
*Hafniaceae*	*Edwardsiella*	*E. tarda*	0	0	0
*Morganellaceae*	*Morganella*	*M. morganii*	0	0	0
	*Providencia*	*P. stuartii*	15	0	23

^1^“No data” – This data is not available from the API20E reference database.

Identifying the Scr pathway in a wider range of species offers the opportunity to examine evolutionary conservation of the proteins and regulatory components. On the protein side, the ScrA PTS transporter subunit (456 amino acids) is the most highly conserved across species, ranging from 91% amino acid identity between the *E. coli* and *C. freundii* proteins, to 81% between the *E. coli* and *Yersinia enterocolitica* proteins. The outer-membrane-spanning ScrY porin, the largest of the gene products at 505 amino acids, retains 64% identity between the *E. coli* and *Y. enterocolitica* proteins. The least conserved of the five *scr* gene products is the ScrB fructose-6-phosphate hydrolase enzyme (467 aa), with only 55% conservation between the *E. coli* and *Y. enterocolitica* proteins.

**Figure 5:**
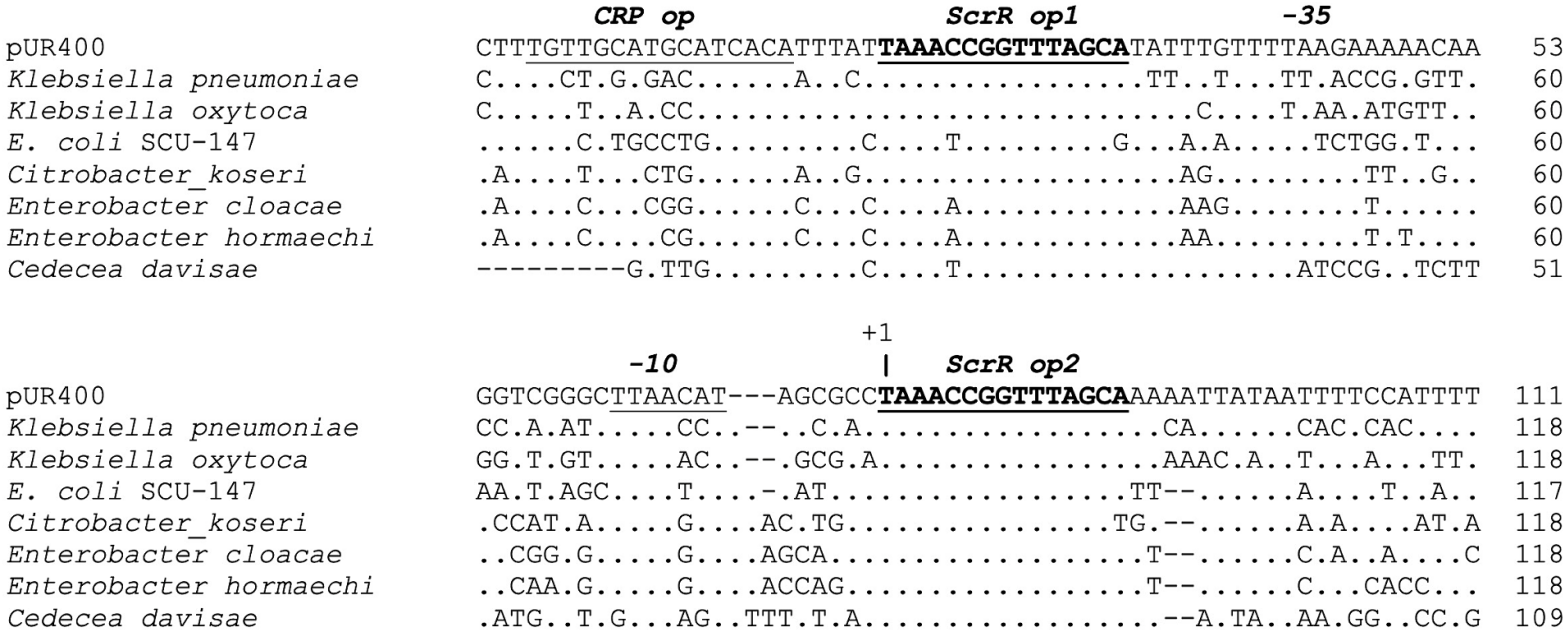
Conservation of the *scrY* promoter and regulatory region. DNA sequences upstream of the *scrY* start codon were aligned using the Geneious alignment algorithm. An identical base at a position is indicated by “.”, and gaps by “−”. Annotations above the aligned sequences are based on Cowan et al. (1991) and Jahreis and Lengeler (1993).

Comparison of non-coding DNA sequences with homologous functions can allow identification of conserved regulatory elements across species. Expression of *scrYAB* is highly inducible by sucrose *in vivo*, with regulation dependent on ScrR, a member of the LacI family of transcriptional regulators. Cowan et al. (1991) and Jahreis and Lengeler (1993) characterized the *scrY* promoter region of pUR400, identifying two 14 bp inverted repeats immediately upstream and downstream of the *scrY* −35 and −10 promoter elements, and providing mutational evidence that these serve as operators for ScrR binding. Jahreis and Lengeler (1993) further showed that, although sucrose induces expression from the *scrY* promoter *in vivo*, ScrR bound to D-fructose or fructose-1-phosphate (products of sucrose degradation) is released from operator DNA to allow RNA polymerase to initiate transcription. Figure 5 shows *scrY* promoter regions from eight species aligned to determine the extent to which the Scr regulatory sequences are conserved across species. The ScrR operator sites are indeed highly conserved sequences in the promoter region. The putative operators are positioned immediately upstream and downstream of the −35 and −10 regions of the promoter, and the downstream operator overlaps directly with the expected transcription start site.

Jahreis and Lengeler (1993) also identified a CRP operator positioned further upstream of the pUR400 *scrY* promoter and demonstrated catabolite repression of this promoter. As seen in Figure 5, the putative CRP operator is not well conserved across species. CRP-mediated catabolite repression of *scr* gene expression therefore may not be conserved in other *Enterobacteriaceae*, but this has not been tested experimentally.

### Analysis of *csc* locus

Most of the literature on sucrose utilization in *E. coli* focuses on the *csc* locus. In our collection of sequenced commensal *E. coli*, seven Suc^+^ isolates contained both the *scr* and *csc* loci, and 15 Suc^+^ isolates contained only the *scr* locus, again demonstrating that it is the *scr* locus that is critical for rapid sucrose utilization. In the absence of the *scr* locus, the presence of the *csc* genes was most strongly associated with the Suc^slow^ phenotype, often with an extended lag phase (see Figure 2C for an example). No *csc*^+^ *scr*^−^ isolates were Suc^+^, but 23 out of 29 were scored as either “slow” or “very slow” for growth on M9 sucrose, including 2 “slow” and 5 “very slow” isolates shown in Table 1. These results are consistent with the literature describing *csc*-dependent sucrose utilization in *E. coli* to be “unusually slow” (Jahreis et al., 2002). Five *csc*^+^ *scr*^−^ isolates [SCU-171 (B2/ST657), 152 (B1/ST54), 483 (B1/ST10955), 106 (B1/ST3695), and 316 (E/ST57)] were completely unable to utilize sucrose for growth. SCU-106 has a 10 bp insertion in the *cscB* coding region, with an accompanying frameshift, but the other isolates have no obvious genetic defect in the *csc* locus to explain the growth deficiencies.

In our contemporary collection, the *csc* locus was found in 37% of isolates. The *csc* locus was universal in phylogroup D and E isolates, nearly universal in B1 isolates, uncommon in B2 isolates, and completely absent in A and F isolates. In the historical ECOR collection, the *csc* locus was present in 25/72 ECOR isolates (35%), similar in frequency to our contemporary collection. The *csc* genes were present in nearly all ECOR B1, D, and E strains (23/27), but rare in B2 strains (2/16), and absent in A and F strains (29 in aggregate).

*csc* resides at a recombination hotspot on the *E. coli* chromosome in close proximity to the *argW* tRNA gene (Jahreis et al., 2002). Moritz and Welch (2006) examined utilization of D-serine by diarrheagenic and uropathogenic *E. coli* and found that D-serine and (slow) sucrose utilization were mutually exclusive, consistent with earlier observations by Alaeddinoglu and Charles (1979). The *cscRAKB* and *dsdCXA* loci typically reside at roughly the same chromosomal location, though there is more than one possible configuration of each locus, perhaps because this is also a frequent target for integration of lambdoid phage. We observed diverse arrangements in this genomic region in our collection, with *dsdC* often retained even when the *csc* locus was present. In a few strains, both the *csc* and *dsd* loci were present in their entirety, but more often isolates had one functional locus or the other, as seen by Moritz and Welch (2006).

The *csc* locus was examined in *Shigella sonnei*, *S. flexneri*, *S. dysenteriae*, and *S. boydii* genomes, and was found to be defective in nearly all cases. Figure 4 compares the wild-type *E. coli csc* locus with loci commonly observed in the genomes of *Shigella* species. The *cscA* and *cdcR* genes were often present and intact, but the *cscK* (fructokinase) and *cscB* (sucrose permease) genes were usually interrupted with *IS1*, *IS3*, or *IS4* elements, or missing altogether. An absence of *scr* loci, combined with disruption of *csc* loci, may explain why utilization/fermentation of sucrose is rare or absent in *Shigella* isolates. *Shigella* genomes rarely contain the *dsd* locus either; only 4% of nearly 2,200 *Shigella* genomes in the RefSeq database contained a full-length, uninterrupted *dsd* locus.

Querying GenBank with the entire *E. coli csc* locus (*Ec_csc* hereafter), only four examples were found in *Escherichia* genomes other than *E. coli*, four examples in coliphage genomes, four in *Citrobacter farmeri* genomes, and one each in a *Citrobacter telavivensis* genome and an *Enterobacter hormaechei* genome. These may have arisen from horizontal gene transfer from *E. coli*. Further inspection also revealed that *Citrobacter freundii* genomes contain a *csc* locus (*Cf_csc* hereafter) that falls below the detection threshold with the *Ec_csc* query in Blastn. *Cf_csc* is clearly homologous to *Ec_csc* when the amino acid sequences of the encoded proteins are compared. The arrangement of genes in *Cf_csc* is altered (Figure 4), with *cscA* and *cscR* each independently inverted. Nearly all (95%) of *C. freundii* RefSeq genomes contain a complete, uninterrupted (and likely functional) *csc* locus with this arrangement. Querying GenBank with *Cf_csc* identified several homologous loci, possibly also resulting from horizontal gene transfer, in *Citrobacter portucalensis*, *Citrobacter youngae*, and *Kluyvera ascorbata* genomes.

**Figure 6:**
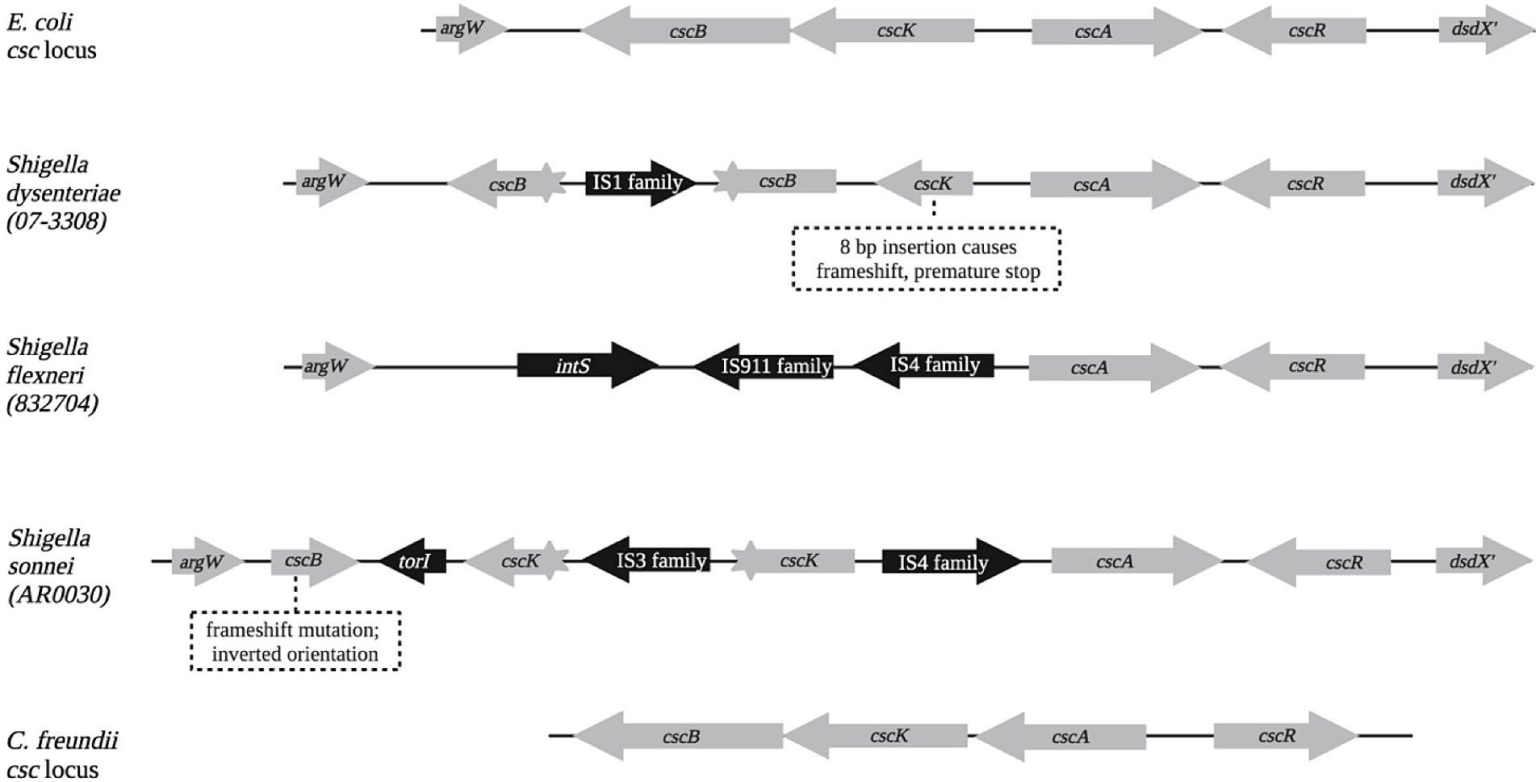
Representative structures of *E. coli*, *Shigella*, and *C. freundii csc* loci. *Shigella* species and strain names are shown on the left side. Note that in the *C. freundii* locus, the *cscA* and *cscR* genes are in the same relative locations, but their orientations are each flipped.

**Figure 7:**
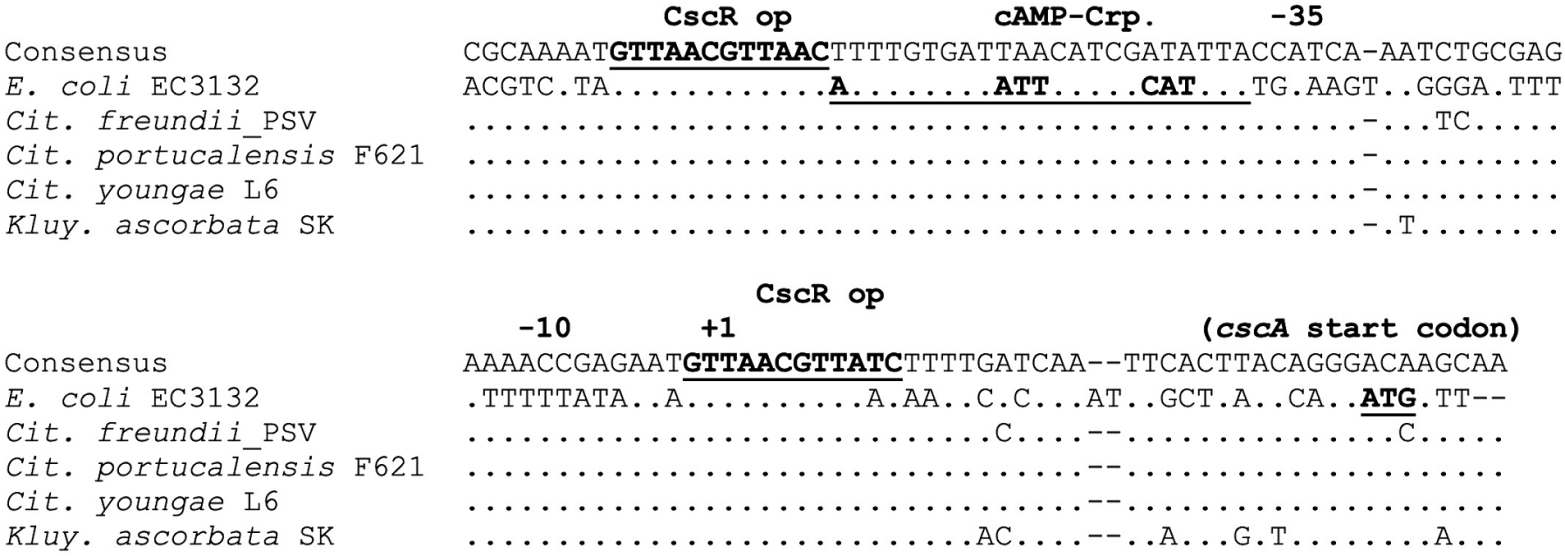
Conservation of the *cscA* promoter and regulatory region. DNA sequences upstream of the *cscA* start codon were aligned using the Geneious alignment algorithm. Identity with the consensus sequence is indicated by “.”, and gaps by “−”. Annotations above the aligned sequences are based on Jahreis et al. (2002).

As with *scr*, identifying the *csc* locus in multiple species allowed comparison of the promoter regions to search for potentially conserved regulatory elements. In *E. coli*, expression of *cscA* is inducible by sucrose *in vivo* and dependent on CscR, which like ScrR is a LacI family member. Jahreis et al. (2002) identified two copies of a 12 bp inverted repeat in the intergenic region between *cscA* and *cscK*; one of these putative operators was the site of a mutation that upregulated expression of *cscA*. The promoter regions upstream of the *cscA* gene in five species were aligned and compared (Figure 6). The inverted repeats identified as potential CscR operators were indeed conserved between *E. coli* and the other species. Assuming a similar functional organization to the *E. coli cscA* promoter, these operators flank either side of the −35 and −10 regions of the promoter, with the downstream operator overlapping the transcriptional start site. Jahreis et al. (2002) also identified a putative Crp-cAMP operator, conventionally positioned adjacent to the −35 region of the *cscA* promoter and demonstrated catabolite repression of the Csc pathway. Extensive conservation of the putative Crp operator is seen between species (Figure 7).

## Discussion

The results presented here support several key findings: (1) a native Scr pathway allows many *E. coli* strains to grow rapidly on sucrose, while the Csc pathway supports slower growth; (2) the Scr and Csc pathways are relatively common and independent in *E. coli*, and sometimes coexist; (3) the Scr and Csc pathways are generally chromosomally encoded, but are occasionally mobilized by non-chromosomal elements; and (4) the Scr pathway is widespread but not universal in *Enterobacteriaceae*, while the Csc pathway is largely confined to *E. coli*, *Shigella* (where it is non-functional), and *Citrobacter* species.

Because the *E. coli* K-12 strains (phylogroup A, ST10) that have been the dominant laboratory models lack both the *scr* and *csc* loci and thus do not utilize sucrose, exploration of sucrose metabolism in *E. coli* has been limited. Prior to this work there were only two reports describing chromosomally-encoded *scr* loci in *E. coli*, in uropathogenic phylogroup B2 ST92 isolate *E. coli* 536 (Brzuszkiewicz et al., 2006), and in enterohemorrhagic phylogroup F isolates (Treviño-Quintanilla et al., 2007). Phylogroup F represents a small fraction of the human-associated *E. coli* in most studies, but B2 strains are more common (e.g., Marin et al., 2022), and B2 isolates carrying *scr* represented nearly 20% of the *E. coli* in our commensal collection, and an even larger fraction among UTI isolates (data not shown).

Some (rare) non-B2 *E. coli* isolates appear to contain *scr* due to horizontal gene transfer (HGT), such as three isolates we identified containing *scr* loci mobilized from *Citrobacter freundii*. In SCU-391, *Cf-scr* is surrounded by prophage genes, suggesting that *scr* could have moved by phage transduction. Other HGT possibilities not seen in our collection include mobilization of *scr* by recombination onto large plasmids, as with the pUR400 plasmid found in the original example of *scr* mobilization from *Salmonella* into *E. coli*. Another possibility is a conjugative transposon, as with CTn*scr94* in *Salmonella senftenburg* (Hochhut et al., 1997). Nevertheless, Smith and Parsell (1975), showed that only three out of 152 sucrose-using *E. coli* strains could transmit the phenotype by conjugation, so *scr* movement by plasmids or conjugative transposons is probably not common in *E. coli*.

The Scr pathway is present in both Gram-negative and Gram-positive bacteria (Reid and Abratt, 2005), and is widespread in the family *Enterobacteriaceae* (Table 2). An upper bound for the evolutionary age of a functioning bacterial Scr system may be the evolution in higher plants of the capacity to synthesize sucrose as a photosynthesis-derived product for carbon and energy storage (Ruan, 2014). Variability across bacterial lineages may reflect erratic selection pressure due to the variability of sucrose abundance in various niches. An association with plants may be more likely to favor this capacity; the gram-negative phytopathogen *Erwinia amylovora*, for example, requires the *scr* locus as a key virulence factor (Bogs and Geider, 2000).

The *csc* locus was the first set of *E. coli* genes experimentally associated with sucrose utilization (Bockmann et al., 1992). Previous genomic analyses showed that *csc* was mutually exclusive with the *dsd* locus for D-serine utilization, which generally resides at the same location on the chromosome. Some authors have speculated that the *dsd* locus is selected for in uropathogenic *E. coli*, as D-serine (but not sucrose) is present in significant concentrations in urine and may serve as both signal and growth substrate (Roesch et al., 2003). Conversely, intestinal pathogenic *E. coli* may not encounter D-serine, but may be selected for utilization of carbohydrates such as sucrose. That rationale must be reconsidered, as the *scr* locus allows *E. coli* isolates to utilize sucrose regardless of whether the *csc* genes are present. Furthermore, we see no significant difference in the frequency of the *dsd*, *csc*, and *scr* genes between the genomes of commensal and uropathogenic *E. coli* in our lab (data not shown).

The appearance of the *csc* locus in *E. coli* must have been prior to divergence of contemporary *Shigella* species, as many *Shigella* genomes have retained the *csc* locus, albeit in non-functional form. Ubiquitous destruction of the *csc* locus, eliminating the ability to utilize sucrose, suggests that this phenotype has been actively selected against in the pathogenic context that shaped *Shigella* evolution. However, a functional *dsd* locus has not replaced *csc* in *Shigellae*, suggesting that metabolism of D-serine is likewise not advantageous for these species.

In conclusion, the Scr pathway is highly effective in supporting rapid growth of *E. coli* with sucrose as a substrate. Little effort toward physiological analysis and/or genetic manipulation of the *E. coli* Scr pathway has been applied to optimize it for bioindustrial applications, particularly relative to what has been done with the Csc pathway. Wider recognition of the significance of the Scr pathway in *E. coli* and other *Enterobacteriaceae* could encourage research on exploitation of sucrose as a feedstock for bacterially-based processes.

## Data availability statement

The original contributions presented in the study are included in the article/supplementary material, further inquiries can be directed to the corresponding author.

## Author contributions

CS: Conceptualization, Data curation, Formal analysis, Funding acquisition, Investigation, Methodology, Project administration, Resources, Supervision, Validation, Writing – original draft, Writing – review & editing. MM: Investigation, Writing – original draft, Writing – review & editing. VL: Investigation, Visualization, Writing – original draft, Writing – review & editing. JJ: Investigation, Visualization, Writing – original draft, Writing – review & editing. AM: Data curation, Investigation, Writing – original draft, Writing – review & editing.

## References

[ref1] Alaeddinoglu N. G. Charles H. P. (1979). Transfer of a gene for sucrose utilization into *Escherichia coli* K12, and consequent failure of expression of genes for D-serine utilization. Microbiology 110, 47–59. 10.1099/00221287-110-1-47 372492

[ref2] Altschul S. F. Gish W. Miller W. Myers E. W. Lipman D. J. (1990). Basic local alignment search tool. J. Mol. Biol. 215, 403–410. doi: 10.1016/S0022-2836(05)80360-2 2231712

[ref3] Aulkemeyer P. Ebner R. Heilenmann G. Jahreis K. Schmid K. Wrieden S.. (1991). Molecular analysis of two fructokinases involved in sucrose metabolism of enteric bacteria. Mol. Microbiol. 5, 2913–2922. doi: 10.1111/j.1365-2958.1991.tb01851.x 1809835

[ref4] Bockmann J. Heuel H. Lengeler J. W. (1992). Characterization of a chromosomally encoded, non-PTS metabolic pathway for sucrose utilization in *Escherichia coli* EC3132. Mol. Gen. Genet. MGG 235, 22–32. doi: 10.1007/BF00286177, PMID: 1435727

[ref5] Bogs J. Geider K. (2000). Molecular analysis of sucrose metabolism of *Erwinia amylovora* and influence on bacterial virulence. J. Bacteriol. 182, 5351–5358. doi: 10.1128/JB.182.19.5351-5358.2000, PMID: 10986236 PMC110976

[ref6] Bruschi M. Boyes S. J. Sugiarto H. Nielsen L. K. Vickers C. E. (2012). A transferable sucrose utilization approach for non-sucrose-utilizing *Escherichia coli* strains. Biotechnol. Adv. 30, 1001–1010. doi: 10.1016/j.biotechadv.2011.08.019, PMID: 21907272

[ref7] Brzuszkiewicz E. Brüggemann H. Liesegang H. Emmerth M. Ölschläger T. Nagy G.. (2006). How to become a uropathogen: comparative genomic analysis of extraintestinal pathogenic *Escherichia coli* strains. Proc. Natl. Acad. Sci. U. S. A. 103, 12879–12884. doi: 10.1073/pnas.0603038103, PMID: 16912116 PMC1568941

[ref8] Chang A. Y. Chau V. Landas J. A. Pang Y. (2017). Preparation of calcium competent *Escherichia coli* and heat-shock transformation. JEMI Methods 1, 22–25.

[ref9] Chen I. M. A. Chu K. Palaniappan K. Ratner A. Huang J. Huntemann M.. (2023). The IMG/M data management and analysis system v. 7: content updates and new features. Nucleic Acids Res. 51, D723–D732. doi: 10.1093/nar/gkac976, PMID: 36382399 PMC9825475

[ref10] Cowan P. J. Nagesha H. Leonard L. Howard J. L. Pittard A. J. (1991). Characterization of the major promoter for the plasmid-encoded sucrose genes *scrY*, *scrA*, and *scrB*. J. Bacteriol. 173, 7464–7470. doi: 10.1128/jb.173.23.7464-7470.1991 1938944 PMC212511

[ref11] Denamur E. Clermont O. Bonacorsi S. Gordon D. (2021). The population genetics of pathogenic *Escherichia coli*. Nat. Rev. Microbiol. 19, 37–54. doi: 10.1038/s41579-020-0416-x 32826992

[ref12] Díez-Villaseñor C. Almendros C. García-Martínez J. Mojica F. J. (2010). Diversity of CRISPR loci in *Escherichia coli*. Microbiology 156, 1351–1361. doi: 10.1099/mic.0.036046-0 28206910

[ref13] Durfee T. Nelson R. Baldwin S. Plunkett G. III Burland V. Mau B.. (2008). The complete genome sequence of *Escherichia coli* DH10B: insights into the biology of a laboratory workhorse. J. Bacteriol. 190, 2597–2606. doi: 10.1128/JB.01695-07, PMID: 18245285 PMC2293198

[ref14] García J. L. (1985). Cloning in *Escherichia coli* and molecular analysis of the sucrose system of the *Salmonella* plasmid SCR-53. Mol. Gen. Genet. MGG 201, 575–577. doi: 10.1007/BF00331358, PMID: 3911031

[ref15] Hardesty C. Ferran C. DiRienzo J. M. (1991). Plasmid-mediated sucrose metabolism in *Escherichia coli*: characterization of *scrY*, the structural gene for a phosphoenolpyruvate-dependent sucrose phosphotransferase system outer membrane porin. J. Bacteriol. 173, 449–456. doi: 10.1128/jb.173.2.449-456.1991, PMID: 1846143 PMC207032

[ref16] Hochhut B. Jahreis K. Lengeler J. W. Schmid K. (1997). CTnscr94, a conjugative transposon found in enterobacteria. J. Bacteriol. 179, 2097–2102. doi: 10.1128/jb.179.7.2097-2102.1997 9079891 PMC178942

[ref17] Holmes B. Willcox W. R. Lapage S. P. (1978). Identification of *Enterobacteriaceae* by the API 20E system. J. Clin. Pathol. 31, 22–30. doi: 10.1136/jcp.31.1.22, PMID: 342546 PMC476713

[ref18] Jahreis K. Bentler L. Bockmann J. Hans S. Meyer A. Siepelmeyer J.. (2002). Adaptation of sucrose metabolism in the *Escherichia coli* wild-type strain EC3132. J. Bacteriol. 184, 5307–5316. doi: 10.1128/JB.184.19.5307-5316.2002, PMID: 12218016 PMC135337

[ref19] Jahreis K. Lengeler J. W. (1993). Molecular analysis of two ScrR repressors and of a ScrR–FruR hybrid repressor for sucrose and D-fructose specific regulons from enteric bacteria. Mol. Microbiol. 9, 195–209. doi: 10.1111/j.1365-2958.1993.tb01681.x, PMID: 8412665

[ref20] Johnson M. Zaretskaya I. Raytselis Y. Merezhuk Y. McGinnis S. Madden T. L. (2008). NCBI BLAST: a better web interface. Nucleic Acids Res. 36, W5–W9. doi: 10.1093/nar/gkn201, PMID: 18440982 PMC2447716

[ref21] Larsen M. V. Cosentino S. Rasmussen S. Friis C. Hasman H. Marvig R. L.. (2012). Multilocus sequence typing of total-genome-sequenced bacteria. J. Clin. Microbiol. 50, 1355–1361. doi: 10.1128/JCM.06094-11, PMID: 22238442 PMC3318499

[ref22] Le Bouguénec C. Schouler C. (2011). Sugar metabolism, an additional virulence factor in enterobacteria. Int. J. Med. Microbiol. 301, 1–6. doi: 10.1016/j.ijmm.2010.04.021 20705507 PMC7129668

[ref23] Lengeler J. W. Mayer R. J. Schmid K. (1982). Phosphoenolpyruvate-dependent phosphotransferase system enzyme III and plasmid-encoded sucrose transport in *Escherichia coli* K-12. J. Bacteriol. 151, 468–471. doi: 10.1128/jb.151.1.468-471.1982, PMID: 7045081 PMC220262

[ref25] Marin J. Clermont O. Royer G. Mercier-Darty M. Decousser J. W. Tenaillon O.. (2022). The population genomics of increased virulence and antibiotic resistance in human commensal *Escherichia coli* over 30 years in France. Appl. Environ. Microbiol. 88, e00664–e00622. 35862685 10.1128/aem.00664-22 PMC9361829

[ref26] Moritz R. L. Welch R. A. (2006). The *Escherichia coli argW-dsd*CXA genetic island is highly variable, and *E. coli* K1 strains commonly possess two copies of *dsdCXA*. J. Clin. Microbiol. 44, 4038–4048. doi: 10.1128/JCM.01172-06, PMID: 17088369 PMC1698345

[ref27] Mukherjee S. Stamatis D. Li C. T. Ovchinnikova G. Bertsch J. Sundaramurthi J. C.. (2023). Twenty-five years of Genomes OnLine Database (GOLD): data updates and new features in v. 9. Nucleic Acids Res. 51, D957–D963. doi: 10.1093/nar/gkac974, PMID: 36318257 PMC9825498

[ref28] Ochman H. Selander R. K. (1984). Standard reference strains of *Escherichia coli* from natural populations. J. Bacteriol. 157, 690–693. doi: 10.1128/jb.157.2.690-693.1984, PMID: 6363394 PMC215307

[ref29] Palchaudhuri S. Rahn S. Santos D. S. Maas W. K. (1977). Characterization of plasmids in a sucrose-fermenting strain of *Escherichia coli*. J. Bacteriol. 130, 1402–1403. doi: 10.1128/jb.130.3.1402-1403.1977, PMID: 324988 PMC235375

[ref30] Peters S. Rose T. Moser M. (2010). Sucrose: a prospering and sustainable organic raw material. Top. Curr. Chem. 294, 1–23. doi: 10.1007/128_2010_58 21626746

[ref31] Pitout J. D. Peirano G. Chen L. DeVinney R. Matsumura Y. (2022). *Escherichia coli* ST1193: following in the footsteps of *E. coli* ST131. Antimicrob. Agents Chemother. 66, e00511–e00522. 35658504 10.1128/aac.00511-22 PMC9295538

[ref32] Pruitt K. D. Tatusova T. Maglott D. R. (2007). NCBI reference sequences (RefSeq): a curated non-redundant sequence database of genomes, transcripts and proteins. Nucleic Acids Res. 35, D61–D65. doi: 10.1093/nar/gkl842 17130148 PMC1716718

[ref33] Reid S. J. Abratt V. R. (2005). Sucrose utilisation in bacteria: genetic organisation and regulation. Appl. Microbiol. Biotechnol. 67, 312–321. doi: 10.1007/s00253-004-1885-y, PMID: 15660210

[ref34] Roesch P. L. Redford P. Batchelet S. Moritz R. L. Pellett S. Haugen B. J.. (2003). Uropathogenic *Escherichia coli* use D-serine deaminase to modulate infection of the murine urinary tract. Mol. Microbiol. 49, 55–67. doi: 10.1046/j.1365-2958.2003.03543.x, PMID: 12823810

[ref35] Ruan Y. L. (2014). Sucrose metabolism: gateway to diverse carbon use and sugar signaling. Annu. Rev. Plant Biol. 65, 33–67. doi: 10.1146/annurev-arplant-050213-040251, PMID: 24579990

[ref36] Sabri S. Nielsen L. K. Vickers C. E. (2013). Molecular control of sucrose utilization in *Escherichia coli* W, an efficient sucrose-utilizing strain. Appl. Environ. Microbiol. 79, 478–487. doi: 10.1128/AEM.02544-12, PMID: 23124236 PMC3553775

[ref37] Schmid K. Ebner R. Altenbuchner J. Schmitt R. Lengeler J. W. (1988). Plasmid-mediated sucrose metabolism in *Escherichia coli* K12: mapping of the *scr* genes of pUR400. Mol. Microbiol. 2, 1–8. doi: 10.1111/j.1365-2958.1988.tb00001.x, PMID: 2835584

[ref38] Schmid K. Ebner R. Jahreis K. Lengeler J. W. Titgemeyer F. (1991). A sugar-specific porin, ScrY, is involved in sucrose uptake in enteric bacteria. Mol. Microbiol. 5, 941–950. doi: 10.1111/j.1365-2958.1991.tb00769.x, PMID: 1649946

[ref39] Schmid K. Schupfner M. Schmitt R. (1982). Plasmid-mediated uptake and metabolism of sucrose by *Escherichia coli* K-12. J. Bacteriol. 151, 68–76. doi: 10.1128/jb.151.1.68-76.1982, PMID: 6211435 PMC220195

[ref40] Schülein K. Schmid K. Benzl R. (1991). The sugar-specific outer membrane channel ScrY contains functional characteristics of general diffusion pores and substrate-specific porins. Mol. Microbiol. 5, 2233–2241. doi: 10.1111/j.1365-2958.1991.tb02153.x 1722560

[ref41] Smith H. W. Parsell Z. (1975). Transmissible substrate-utilizing ability in enterobacteria. Microbiology 87, 129–140. 10.1099/00221287-87-1-129 1094091

[ref42] Sprenger G. A. Lengeler J. W. (1988). Analysis of sucrose catabolism in *Klebsiella pneumoniae* and in Scr+ derivatives of *Escherichia coli* K12. Microbiology 134, 1635–1644. doi: 10.1099/00221287-134-6-1635, PMID: 3065452

[ref43] Stephens C. Arismendi T. Wright M. Hartman A. Gonzalez A. Gill M.. (2020). F plasmids are the major carriers of antibiotic resistance genes in human-associated commensal *Escherichia coli*. MSphere 5, 10–1128. 10.1128/mSphere.00709-20 PMC7407071 32759337

[ref44] The H. C. Thanh D. P. Holt K. E. Thomson N. R. Baker S. (2016). The genomic signatures of *Shigella* evolution, adaptation and geographical spread. Nat. Rev. Microbiol. 14, 235–250. doi: 10.1038/nrmicro.2016.10, PMID: 26923111

[ref45] Titgemeyer F. Jahreis K. Ebner R. Lengeler J. W. (1996). Molecular analysis of the *scrA* and *scrB* genes from *Klebsiella pneumoniae* and plasmid pUR400, which encode the sucrose transport protein enzyme II Scr of the phosphotransferase system and a sucrose-6-phosphate invertase. Mol. Gen. Genet. MGG 250, 197–206. 8628219 10.1007/BF02174179

[ref46] Treviño-Quintanilla L. G. Escalante A. Caro A. D. Martínez A. González R. Puente J. L.. (2007). The phosphotransferase system-dependent sucrose utilization regulon in enteropathogenic *Escherichia coli* strains is located in a variable chromosomal region containing iap sequences. Microbial Physiol. 13, 117–125. doi: 10.1159/000103603 17693719

[ref47] Wohlhieter J. A. Lazere J. R. Snellings N. J. Johnson E. M. Synenki R. M. Baron L. S. (1975). Characterization of transmissible genetic elements from sucrose-fermenting *Salmonella* strains. J. Bacteriol. 122, 401–406. doi: 10.1128/jb.122.2.401-406.1975, PMID: 1092649 PMC246070

